# Evidence of a metacognitive illusion in stimulus-specific prospective judgments of distraction by background speech

**DOI:** 10.1038/s41598-024-74719-4

**Published:** 2024-10-15

**Authors:** Gesa Fee Komar, Axel Buchner, Laura Mieth, Ruben van de Vijver, Raoul Bell

**Affiliations:** 1 Department of Experimental Psychology, Heinrich Heine University Düsseldorf, Düsseldorf, Germany; 2 Department of Linguistics, Heinrich Heine University Düsseldorf, Düsseldorf, Germany

**Keywords:** Psychology, Human behaviour

## Abstract

Two experiments served to examine how people arrive at stimulus-specific prospective judgments about the distracting effects of speech on cognitive performance. The direct-access account implies that people have direct metacognitive access to the cognitive effects of sounds that determine distraction. The processing-fluency account implies that people rely on the processing-fluency heuristic to predict the distracting effects of sounds on cognitive performance. To test these accounts against each other, we manipulated the processing fluency of speech by playing speech forward or backward and by playing speech in the participants’ native or a foreign language. Forward speech and native speech disrupted serial recall to the same degree as backward speech and foreign speech, respectively. However, the more fluently experienced forward speech and native speech were incorrectly predicted to be less distracting than backward speech and foreign speech. This provides evidence of a metacognitive illusion in stimulus-specific prospective judgments of distraction by speech, supporting the processing-fluency account over the direct-access account. The difference between more and less fluently experienced speech was largely absent in the participants’ global retrospective judgments of distraction, suggesting that people gain access to comparatively valid cues when experiencing the distracting effects of speech on their serial-recall performance firsthand.

With the increasing use of portable devices such as smartphones, tablets and laptops, individuals often undertake cognitively demanding tasks in shared spaces, amidst the everyday noise that accompanies these communal settings. Specifically, background conversations are a source of distraction in private and public shared spaces like living rooms, open-plan offices, cafés and public transport. Not only is background speech present in many working and learning environments, but it also has been identified as one of the most distracting sounds in research on auditory distraction^1,2^. As a potential remedy, technological advancement allows people to control their auditory environment, for example, by using noise-canceling headphones without playing any sounds from the device to which the headphones are attached. In order to understand whether people will control their auditory environment appropriately, it is important to examine how people arrive at metacognitive judgments of distraction. For instance, the metacognitive judgment about whether background conversations will interfere with learning may determine whether a student will use noise-canceling headphones to block out the roommates’ conversation while studying for an exam. However, can people, after they have listened to speech, correctly predict the distracting effect the speech will have on cognitive performance? This question is addressed in the present study.

Judgments of distraction refer to the degree to which task-irrelevant stimuli affect cognitive performance. Judgments of learning ^3–11^, by contrast, refer to the processing and retention of task-relevant stimuli. Judgments of distraction can thus be seen as the counterpart to judgments of learning. Whereas judgments of learning have been in the focus of research on metacognition for some decades, judgments of distraction are only now receiving increasing attention in research^12–17^. In these studies, judgments of distraction pertained to the effects of sounds on performance in a serial-recall task, an established experimental paradigm to assess effects of auditory distraction (for a review, see^2^). In two of these studies^14,16^, different types of judgments of distraction were measured, following procedures developed for assessing judgments of learning^3,4,7,8^. Participants made *stimulus-specific prospective judgments* in response to specific sounds, knowing that these sounds would later have to be ignored while memorizing sequences of digits for serial recall. Participants listened to each sound individually. Immediately after the presentation of each sound, they were asked to predict how distracting or helpful this sound would be, relative to quiet, for serial recall. The task of providing stimulus-specific prospective judgments serves as a model task for the everyday situation in which people make judgments about the distracting effects of sounds on cognitive performance based on the immediate experience of the sounds. For example, when deciding on whether it is acceptable to study in the living room where roommates are talking in the background, a student may briefly listen to the auditory background and then decide whether the conversation is so distracting that it will interfere with studying. Furthermore, after participants had gained firsthand experience with the distracting effects of the sounds on their serial-recall performance, they made *global retrospective judgments*. Specifically, participants judged how distracting or helpful the different types of sound had been for serial recall. The task of providing global retrospective judgments serves as a model task for the everyday situation in which people make judgments about the distracting effects of different types of sound on their cognitive performance based on multiple past experiences. For example, after having studied several times in the living room while roommates were talking, a student may no longer feel the need to listen to this specific auditory background in order to decide whether it will interfere with studying—instead, the student may make this decision based on a global judgment of the past experience. In the following, we will develop hypotheses regarding the stimulus-specific prospective judgments, but we will return to the global retrospective judgments at the end of the introduction.

Two competing theoretical accounts have been put forward to explain how people arrive at stimulus-specific prospective judgments of distraction^14,16^. According to the *direct-access account*, people have direct metacognitive access to the cognitive effects of sounds that determine distraction, allowing them to correctly predict the distracting effects of speech on cognitive performance. It is often assumed that attention is closely linked to awareness. For example, within the embedded-processes model of working memory^18,19^, “the focus of attention is assumed to be the same as the contents of conscious awareness” (^18^, p. 200). Therefore, it is possible that people are directly aware of the degree to which different types of sound attract their attention, thus distracting them when played during a serial-recall task. According to the *processing-fluency account*^16^, by contrast, people rely on the processing-fluency heuristic when making stimulus-specific prospective judgments of distraction, similar to how they make stimulus-specific prospective judgments of learning (e.g.,^3,5,6,9,20,21^). Processing fluency refers to the *subjective* experience of ease or difficulty of processing a stimulus, such as an overheard sound. For instance, people might naively infer that a sound that is experienced as being relatively easy to process requires comparatively little cognitive effort, thereby leaving more cognitive resources available for tasks such as memorizing sequences of digits, compared to when a sound is experienced as being relatively difficult to process. People should therefore predict that sounds which evoke a subjective experience of relative fluency will have smaller distracting effects on serial-recall performance than sounds which evoke a subjective experience of relative disfluency.

In line with previous research^22,23^, we conceive of processing fluency as a subjective experience and we treat its correspondence to the objective difficulty of processing—as measured by distraction in the present study—as an empirically open question. The processing-fluency heuristic may well have some ecological validity^24^. Nevertheless, specific situations can be identified in which the subjective experience of processing fluency should lead to invalid stimulus-specific prospective judgments of distraction. These situations are attractive for research because they allow dissociating the effects of the perceived ease or difficulty of processing from the objective effects of sounds on cognitive performance. If the processing-fluency account is accurate, then individuals will rely on their subjective experience of processing fluency, leading them to make stimulus-specific prospective judgments that, in these specific situations, are inconsistent with the objective effects of the sounds on cognitive performance. By contrast, if the direct-access account is accurate, individuals will be able to directly access the degree to which different types of sound cause distraction, leading them to make stimulus-specific prospective judgments that are consistent with the objective effects of the sounds on cognitive performance. By identifying these specific situations, it is thus possible to test the direct-access account against the processing-fluency account.

So far, the results favor the processing-fluency account over the direct-access account because it can be demonstrated that people sometimes fail to correctly predict the distracting effects of sounds on cognitive performance^25–27^. A direct test of the competing accounts has been provided by Bell et al.^16^. In that study, processing fluency was manipulated by playing instrumental music—piano melodies and segments of Mozart’s sonata K. 448—either in the original forward direction or in backward direction. A separate norming study had shown that the subjective experience of processing fluency was higher for forward than for backward music. By contrast, it is well known that the playback direction of instrumental music does not affect its objective distraction effect on serial-recall performance^28,29^. This is so because the number of changes in the to-be-ignored auditory channel and the acoustic complexity of the sounds—and thus the acoustic properties that are primarily responsible for auditory distraction in serial recall^1,2^—are held constant across both conditions. Bell et al.^16^ calculated the objective distraction effects by subtracting, from the mean number of digits per trial recalled correctly in each of the two variants of the distractor trials (with forward or backward music), the mean number of digits per trial recalled correctly in the quiet control trials. The results showed that forward music disrupted serial recall to the same degree as backward music. While participants correctly predicted that backward music would be distracting relative to quiet, they incorrectly predicted that forward music would not be distracting. This metacognitive illusion in the stimulus-specific prospective judgments of distraction favors the processing-fluency account over the direct-access account.

The processing-fluency account was offered as a general explanation of how people arrive at stimulus-specific prospective judgments of distraction, including stimulus-specific prospective judgments of distraction by speech, but so far the metacognitive illusion has only been demonstrated by contrasting forward with backward music^16^. It is important to also investigate whether there is a metacognitive illusion when the distracting sounds consist of speech rather than instrumental music. This is so because people are reasonably good at predicting the relative size of the distracting effects of repeated, deviating and changing speech on serial-recall performance^14^. Specifically, repeated speech (i.e., a sequence of identical speech sounds) is predicted to be less distracting than deviating speech (i.e., a sequence of speech sounds in which one speech sound differs from the other identical speech sounds) which, in turn, is predicted to be less distracting than changing speech (i.e., a sequence of speech sounds in which all speech sounds differ from one another). Consistent with these predictions, repeated speech causes less distraction in serial recall than deviating speech which, in turn, causes less distraction in serial recall than changing speech. This pattern of findings is in line with the processing-fluency account^16^. However, the same pattern of findings would be expected if people were better at predicting the distracting effects of speech compared to the distracting effects of instrumental music on serial-recall performance. This may be due to greater familiarity with the distracting effects of speech compared to the distracting effects of instrumental music or because speech causes more pronounced distraction in serial recall than instrumental music^1,2^, making it easier to correctly predict the more familiar and more pronounced distracting effects of speech than the less familiar and less pronounced distracting effects of instrumental music. There is indeed some evidence suggesting that people may find it particularly challenging to correctly predict the distracting effects of instrumental music on serial-recall performance^12,27^. Therefore, it is important to test whether a metacognitive illusion in stimulus-specific prospective judgments of distraction can be observed not only with instrumental music^16^ but also with speech.

The experiments reported here were conducted to provide this test of the direct-access account against the processing-fluency account. In Experiment 1, we manipulated the processing fluency of speech sequences by varying their playback direction, parallel to the variation of the playback direction of piano melodies in the study of Bell et al.^16^. Forward speech should give rise to a higher subjective experience of processing fluency than backward speech, an assumption we confirmed in a separate validation study (see below). In contrast, varying the playback direction of speech sequences does not affect the number of auditory changes and the acoustic complexity of the speech sequences. Specifically, the number of auditory changes is the same in forward and backward speech, leading to similar levels of distraction in serial recall due to interference-by-process^30–33 ^or attentional capture^29,34–36^. As a result, forward speech has about the same objective distraction effect on serial-recall performance as backward speech^29,37–40^. Comparing stimulus-specific prospective judgments about the distracting effects of forward and backward speech on serial-recall performance thus makes it possible to test the direct-access account against the processing-fluency account. According to the direct-access account, people have direct metacognitive access to the cognitive effects of sounds that determine distraction, allowing them to correctly predict that forward speech disrupts serial recall to the same degree as backward speech. According to the processing-fluency account, people rely on the processing-fluency heuristic when making stimulus-specific prospective judgments of distraction. Evoking a subjective experience of relative fluency, forward speech should be predicted to be less distracting than backward speech. This implies assuming that the metacognitive illusion found in stimulus-specific prospective judgments of distraction by forward and backward music^16^ generalizes to speech.

For the present Experiment 2, we chose a conceptually similar but more ecologically valid manipulation of processing fluency. Participants were proficient German speakers and the large majority of them reported German to be their native language. Given this sample, we compared the distracting effects of German (henceforth native) speech to Japanese (henceforth foreign) speech, spoken by the same German-Japanese bilingual speaker. Native speech should be experienced as more fluent than foreign speech. However, it is well established that native and foreign speech have similar objective distraction effects on serial-recall performance^37,41–43^, probably because the number of auditory changes is comparable in native and foreign speech, leading to similar levels of distraction in serial recall due to interference-by-process^30–33 ^or attentional capture^29,34–36^. When differences between native and foreign speech were observed, these were small and in the direction of a somewhat more pronounced objective distraction effect in the native-speech condition than in the foreign-speech condition^44^. Again, the direct-access account implies that the stimulus-specific prospective judgments of distraction correctly reflect these objective distraction effects. The processing-fluency account, by contrast, implies that the subjective experience of processing fluency associated with native speech relative to foreign speech leads to a metacognitive illusion in stimulus-specific prospective judgments of distraction. Specifically, participants should incorrectly predict native speech to be less distracting than foreign speech.

In addition to stimulus-specific prospective judgments of distraction, which are critical for testing the direct-access account against the processing-fluency account, the present study also included global retrospective judgments of distraction collected after participants had experienced the distracting effects of more and less fluently experienced speech on their serial-recall performance firsthand. In the study involving forward and backward music^16^, the global retrospective judgments showed that participants failed to correctly judge that forward music had disrupted serial recall to the same degree as backward music, even though participants retrospectively judged forward music to have been somewhat distracting relative to quiet. The firsthand experience of performing the serial-recall task while ignoring the different types of instrumental music thus led participants realize that they were distracted by forward music, suggesting that they gained access to comparatively valid cues for judging the distracting effects of instrumental music on their serial-recall performance. Nevertheless, this firsthand experience could not fully prevent a metacognitive illusion in the global retrospective judgments. In the present study, we extend these findings by examining whether people are able to correctly judge the distracting effects of more and less fluently experienced speech after having experienced the distracting effects of the different types of speech on their serial-recall performance firsthand.

## Experiment 1

### Method

#### Participants

The experiment was implemented online using SoSci Survey^45^. To gain fast access to a large number of participants outside of the typical population of Psychology students at universities, we relied on an online-access panel for data collection. This approach is validated by empirical evidence showing that key effects of auditory distraction successfully replicate in online settings^46^. Participants were recruited via the online-access-panel provider Cint (https://www.cint.com/). Participants had to use a desktop or laptop computer. We aimed to collect at least 250 valid data sets and stopped data collection at the end of the day during which this criterion was reached. Of the 347 participants who had passed the audio check (see below), 77 participants did not complete the experiment or withdrew their consent to the use of their data, three participants were under 18 years old and thus not of legal age in Germany and 11 participants reported studying or having studied Psychology (cf.^16^). As a consequence, 91 data sets could not be included into the final analysis. Based on the response in the catch trial or responses to the post-experiment questions (see below), it would have been possible to exclude data sets of a further 49 participants. Following a recommendation of Elliott et al.^46^, we included these data sets into the final analysis. There was one case in which this decision affected the statistical conclusions which is reported below. The final sample, characterized by diversified levels of education, consisted of *N* = 256 participants (124 female, 132 male) with a mean age of 35 (*SD* = 12) years. All participants were proficient German speakers; 238 indicated that German was their native language. Participants were randomly assigned to either the prospective-judgments group (*n* = 134) or the control group (*n *= 122). A sensitivity analysis^47^ showed that, given a sample size of *N* = 256 and α = 0.05, a main effect of the playback direction of speech on the objective distraction effect of the size $\eta_p^2=0.05$ could be detected with a statistical power of 1 − β = 0.95.

#### Ethics statement

In both experiments reported here, participants gave written informed consent prior to participation. The ethics committee of the Faculty of Mathematics and Natural Sciences at Heinrich Heine University Düsseldorf has approved a series of experiments on metacognition of auditory distraction which includes both experiments reported here. Both experiments were conducted in line with the Declaration of Helsinki.

#### Materials

The sounds consisted of 16 speech sequences that have been demonstrated to produce pronounced auditory distraction in previous studies^29,48–50^. The speech sequences (e.g., “Peel, quarter and slice the onions. Add the tomatoes, then simmer it for a few minutes on medium heat.”; translated from German) were spoken by a male voice. Each sound lasted eight seconds. Depending on the condition, the sound file was left in the original forward direction or the entire sound file was reversed using Amadeus Pro (HairerSoft, Version 2.8.9) so as to play the speech in backward direction. While maintaining the number of auditory changes and the acoustic complexity of the sound, reversing the sound file affects other global and local features of the speech. For instance, it transforms the melodic contour of the speech, inverts formants, affects coarticulation effects and reverses transitions into and away from phonemes. Backward speech should evoke a lower subjective experience of processing fluency than forward speech because it runs counter to conventional auditory processing patterns.

A separate validation study with different participants was performed to test whether the manipulation of processing fluency was successful. After having applied the same exclusion criteria as described above, data sets of *N* = 116 participants (33 female, 82 male, 1 nonbinary) with a mean age of 39 (*SD *= 14) years were included into the final analysis. All participants were proficient German speakers; 112 indicated that German was their native language. In each trial of the validation study, participants started the playback of a sound by pressing a “Play” button. The 32 sounds—16 speech sequences with forward speech, 16 speech sequences with backward speech, presented in a random order—were rated one by one. As a measure of the subjective experience of processing fluency, we used a single-item processing-fluency scale because Graf et al.^51^ have demonstrated that a single-item processing-fluency scale ranging from difficult to easy captures the effects of various manipulations of processing fluency such as visual contrast, typicality, repeated exposure and pronounceability as validly as a multiple-item scale. Participants were asked “How difficult or easy was it for you to listen to the sound?”. They responded using the single-item processing-fluency scale ranging from − 6 (very difficult) to + 6 (very easy). Using the speech sequences as the units of analysis, forward speech (*M* = 4.82, *SD* = 0.37) was rated to be significantly easier to listen to than backward speech (*M* = − 3.39, *SD* = 0.20), *F*(1, 30) = 6182.75, *p* < 0.001, $\eta_p^2>0.99$, which leads to the conclusion that processing fluency was manipulated successfully.

#### Design and procedure

At the beginning of the experiment, participants were instructed to participate alone in a quiet environment without distractions. They were asked to turn off their smartphone and to close any other browser windows and programs on their computer before participating. They were informed that they would need a browser that supported the automatic playback of sounds. They were given the option to view on-screen instructions of how to enable automatic sound playback in different browsers. Participants were asked to wear headphones throughout the experiment. The experiment started with an audio check in which spoken letters had to be identified. In each trial, one letter from the set {D, F, H, J, P, Q, R, S, V, Z} was randomly selected and presented at an intensity that matched the intensity of the speech played in the experiment proper. The letter was spoken by a male voice. The participants had to type the letter they had heard into a text field. They were instructed to adjust the volume of the computer to make sure that they could hear everything clearly. They were asked to adjust the browser settings or to switch browsers if no spoken letters were audible. Participants could only proceed once they had correctly identified five letters in a row. Once participants had completed the audio check, they were instructed not to change the volume of their computer for the entire duration of the experiment.

**Figure 1:**
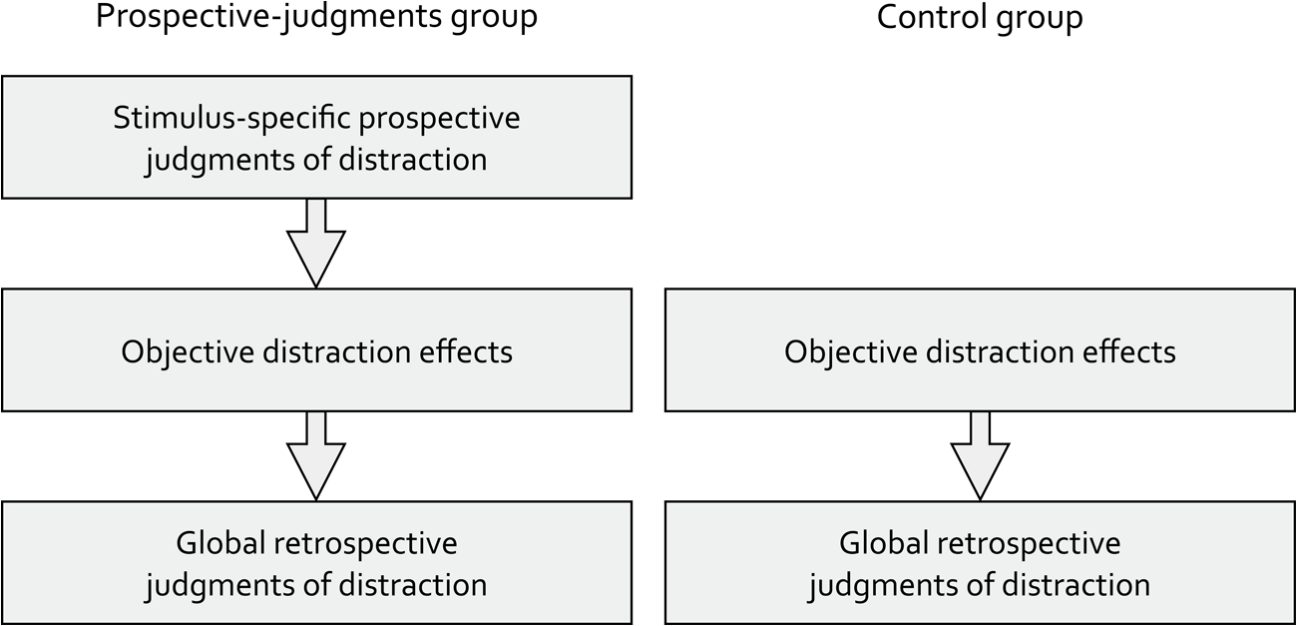
Schematic illustration of the procedure. The procedure comprised three phases in the prospective-judgments group and two phases in the control group. In the prospective-judgments group, participants started the experiment by providing stimulus-specific prospective judgments about the distracting effects which 16 different sounds (eight forward-speech sequences, eight backward-speech sequences) would have on serial-recall performance. To measure the objective distraction effects of speech on serial-recall performance, all participants completed eight quiet training trials and 24 experimental trials of the serial-recall task (eight distractor trials with forward speech, eight distractor trials with backward speech, eight quiet control trials). After participants had experienced the distracting effects of forward and backward speech on their serial-recall performance firsthand, they were asked to provide global retrospective judgments about the distracting effects of the different types of speech on their serial-recall performance.

As in the study of Bell et al.^16^, participants were randomly assigned to two groups (Fig. 1). In the prospective-judgments group, participants were asked to provide stimulus-specific prospective judgments about the effects of speech on serial-recall performance before the objective distraction effects were measured. The control group started directly with the serial-recall task. As in research on judgments of learning^7,11,52^, this experimental design served to explore whether reflecting on the effects of speech on serial-recall performance when making stimulus-specific prospective judgments would have downstream effects on the objective distraction effects in the serial-recall task (e.g.,^12^). This would be the case, for instance, when people feel motivated to put more effort into the serial-recall task after having reflected on the effects of speech on serial-recall performance (but see^13,16^). Finally, all participants were asked to provide global retrospective judgments about the distracting effects of the different types of speech on their serial-recall performance.

*Stimulus-specific prospective judgments of distraction *were collected in the prospective-judgments group before the serial-recall task. Participants were asked to imagine that they were performing a serial-recall task that was described and illustrated with an example trial in quiet (see the next paragraph for a description of the serial-recall task). After the example trial, participants were provided with instructions for the prospective-judgments task. They were informed that various sounds were going to be played to them. They were asked to imagine hearing the sounds while performing the memorization task that had just been demonstrated to them. In each trial of the prospective-judgments task, participants initiated the playback of the sound by clicking a button, whereupon one of the speech sequences was played for eight seconds. One second after the end of the speech sequence, participants were asked to predict how distracting or helpful they thought the sound would be for serial recall relative to quiet on a metacognition scale ranging from − 6 (very distracting) to + 6 (very helpful). The scale contained intermediate verbal labels for the values of − 4 (distracting), − 2 (somewhat distracting), 0 (neither nor), + 2 (somewhat helpful) and + 4 (helpful) to facilitate the interpretation of the metacognition scale^53,54^. For each participant in the prospective-judgments group, eight forward-speech sequences and eight backward-speech sequences were randomly selected from the pool of 16 forward-speech sequences and 16 backward-speech sequences, respectively. The speech sequences were presented in a random order.

*Objective distraction effects* of speech on serial-recall performance were measured after the prospective-judgments task in the prospective-judgments group or at the beginning of the experiment in the control group. Participants were instructed that several trials would follow in which their task was to memorize the order of digits without external aids and without speaking the digits out loud. They were also informed that they would occasionally hear sounds over the headphones while the digits were shown. They were asked to ignore these sounds. Participants were instructed that, after each sequence of digits, they had to recall the digits in the correct order and to guess if they did not remember a digit.

In each trial of the serial-recall task, a sequence of eight digits was generated by randomly drawing digits without replacement from the set {1, 2, …, 9}. Each digit was presented for one second at the center of the browser window. One second after the last digit had been presented, participants had to recall the digits in the order of their presentation by typing eight digits into a text field with eight question marks. Participants had to enter exactly eight digits to continue.

Eight quiet training trials were used to familiarize participants with the task. The 24 experimental trials included eight distractor trials with forward speech, eight distractor trials with backward speech and eight quiet control trials. Speech was played concurrently to the presentation of the sequence of the to-be-remembered digits. The experimental trials were presented in an order that was randomized at an individual level.

For each participant in the prospective-judgments group, the eight forward-speech sequences and the eight backward-speech sequences that had not been presented in the prospective-judgments task were used as distractor sequences in the serial-recall task. For each participant in the control group, eight forward-speech sequences and eight backward-speech sequences were randomly selected from the pool of 16 forward-speech sequences and 16 backward-speech sequences, respectively. In a catch trial at the end of the serial-recall task, the spoken letter “q” was presented and participants were asked to type the letter they had heard into a text field.

*Global retrospective judgments of distraction* were collected from all participants after the serial-recall task had been completed. Participants were informed about the type of sound whose effect on their serial-recall performance they had to judge (“In some trials, you have heard sentences that were played forward” or “In some trials, you have heard sentences that were played backward”). They were asked to judge how distracting or helpful this type of sound had been for serial recall on a scale ranging from − 6 (very distracting) to + 6 (very helpful). The order in which the two types of sound had to be judged was randomly determined.

At the end of the experiment, participants were thanked for their participation. Participants were instructed to provide honest responses to the post-experiment questions “Was all the information presented correctly?” and “Did you follow the instructions?” so that reliable conclusions could be drawn from the results. Participants were told that it would not be disadvantageous for them if they responded “no” (cf.^55^). Directly after these post-experiment questions, participants were asked to confirm their consent to the use of their data, with the opportunity to revoke their consent given at the beginning of the experiment by selecting a “No, I withdraw the consent to the use of my data” option. The median duration of the experiment was 21 minutes in the prospective-judgments group and 15 minutes in the control group.

### Results

#### Stimulus-specific prospective judgments of distraction

**Figure 2:**
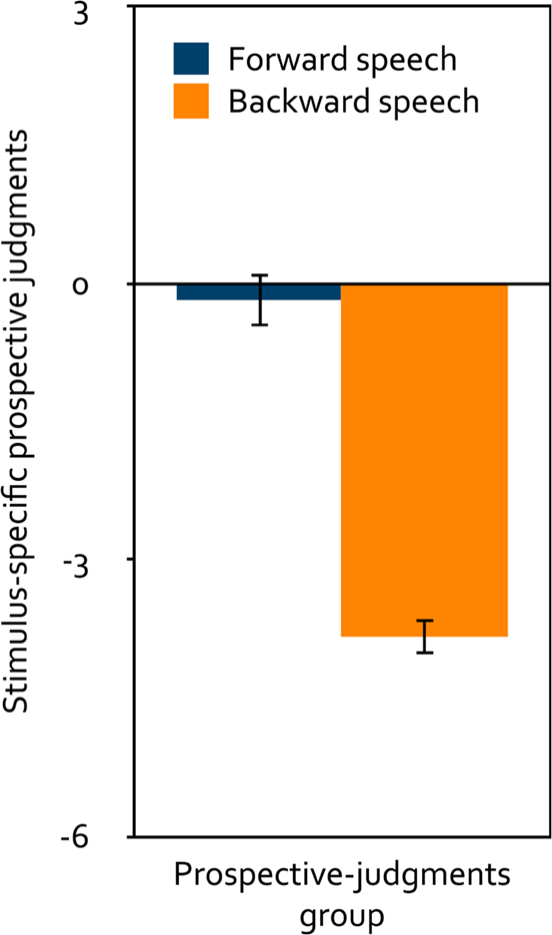
Mean stimulus-specific prospective judgments about the distracting effect of speech on serial-recall performance on a scale ranging from − 6 (very distracting) to + 6 (very helpful) as a function of playback direction (forward, backward) in Experiment 1. The error bars represent the standard errors of the means.

A repeated-measures analysis of variance was calculated to test the effect of the within-subjects factor playback direction (forward, backward) on the stimulus-specific prospective judgments (Fig. 2). Participants in the prospective-judgments group predicted forward speech to be significantly less distracting than backward speech, *F*(1, 133) = 121.52, *p* < 0.001, $\eta_p^2=0.48$. Relative to the neutral midpoint of the scale, forward speech was predicted to be neither distracting nor helpful, *t*(133) = − 0.63, *p* = 0.531, *d* = − 0.05, whereas backward speech was predicted to be distracting, *t*(133) = − 19.79, *p* < 0.001, *d* = − 1.71.

#### Objective distraction effects

The objective distraction effects of speech on serial-recall performance were calculated for each participant by subtracting, from the mean number of digits per trial that were recalled at the correct serial position in each of the two variants of the distractor trials (with forward or backward speech), the mean number of digits per trial that were recalled at the correct serial position in the quiet control trials. The serial-recall results that formed the basis for calculating the objective distraction effects, averaged across participants in each group, are reported in Table 1.

**Table 1:** Mean number of digits per trial that were recalled correctly in each of the two variants of the distractor trials (forward speech, backward speech) and in the quiet control trials of the serial-recall task as a function of group (prospective judgments, control) in Experiment 1. Eight different digits were presented per trial. A strict serial-recall criterion was used in that only digits reproduced at the correct serial position were scored as correct. The values in parentheses represent the standard errors of the means.

	Prospective-judgments group		Control group	
Forward speech	4.60	(0.19)	4.48	(0.19)
Backward speech	4.64	(0.20)	4.49	(0.18)
Quiet control	5.31	(0.18)	5.11	(0.19)

**Figure 3:**
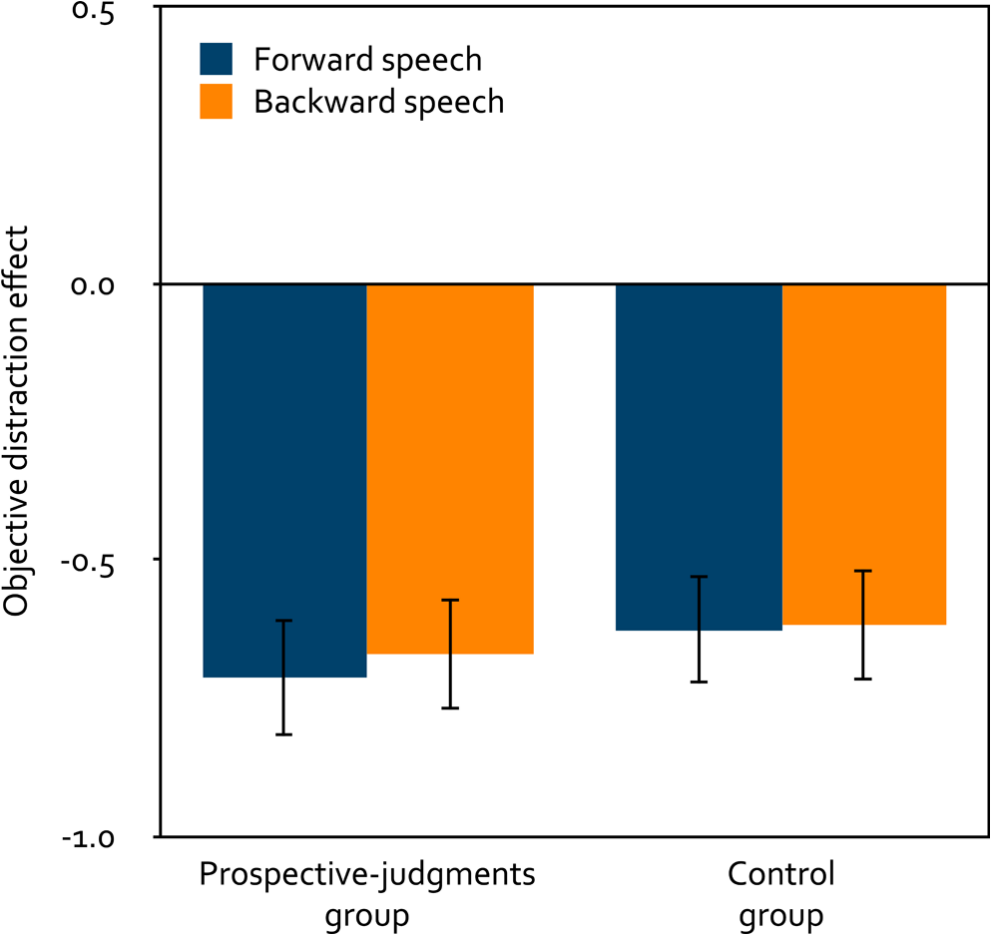
Mean objective distraction effect of speech on serial-recall performance as a function of playback direction (forward, backward) and group (prospective judgments, control) in Experiment 1. The objective distraction effects were calculated by subtracting, from the mean number of digits per trial recalled correctly in the distractor trials, the mean number of digits per trial recalled correctly in the quiet control trials. Negative values thus reflect a distracting effect of the speech (positive values would reflect a helpful effect). The error bars represent the standard errors of the means.

A 2 × 2 mixed analysis of variance was calculated to test the effects of the within-subjects factor playback direction (forward, backward) and the between-subjects factor group (prospective judgments, control) on the objective distraction effect of speech on serial-recall performance (Fig. 3). Forward speech disrupted serial recall just as much as backward speech, *F*(1, 254) = 0.16, *p* = 0.691, $\eta_p^2=\;< 0.01$. Whether or not stimulus-specific prospective judgments had been collected before the serial-recall task did not affect the objective distraction effect, *F*(1, 254) = 0.30, *p* = 0.586, $\eta_p^2< 0.01$, and did not interact with playback direction, *F*(1, 254) = 0.06, *p* = 0.814, $\eta_p^2< 0.01$. In the prospective-judgments group, both forward speech, *t*(133) = − 6.74, *p* < 0.001, *d* = − 0.58, and backward speech, *t*(133) = − 6.70, *p* < 0.001, *d* = − 0.58, had a distracting effect on serial-recall performance relative to quiet. Likewise, in the control group, both forward speech, *t*(121) = − 6.34, *p* < 0.001, *d* = − 0.57, and backward speech, *t*(121) = − 6.08, *p* < 0.001, *d* = − 0.55, had a distracting effect on serial-recall performance relative to quiet.

#### Global retrospective judgments of distraction

**Figure 4:**
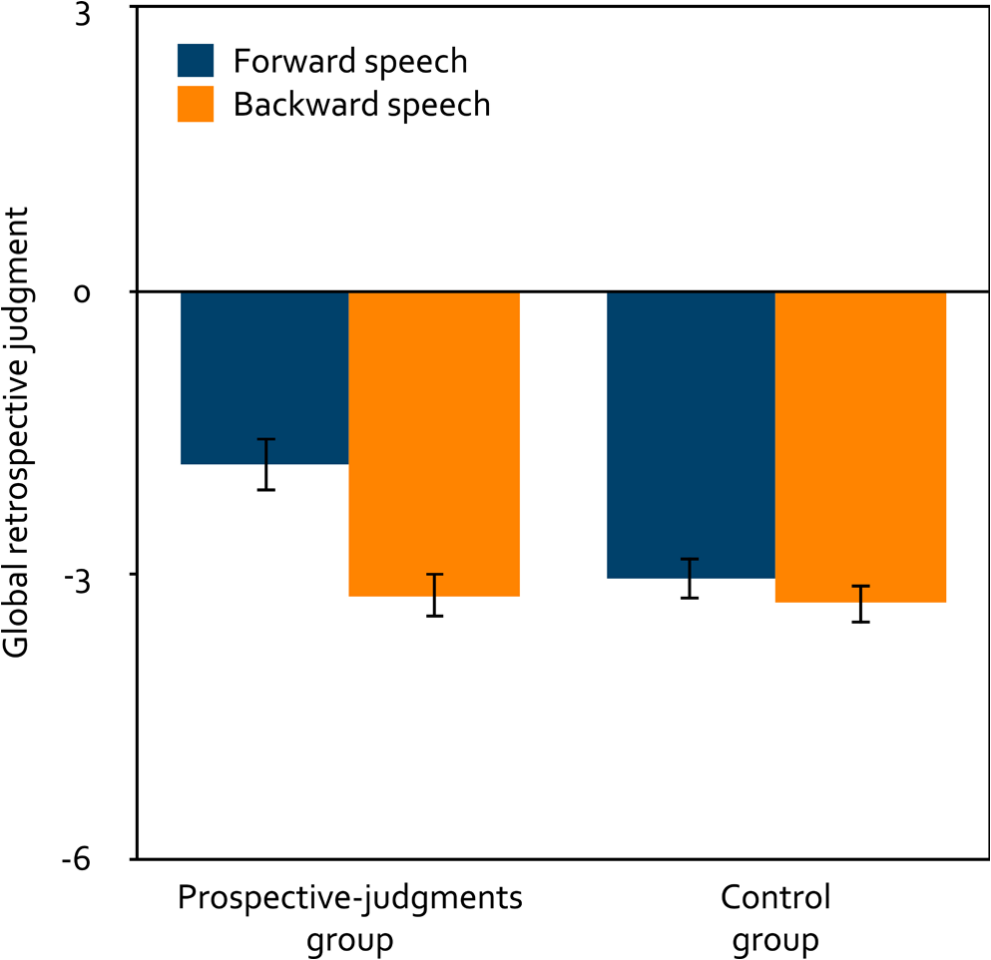
Mean global retrospective judgment about the distracting effect of speech on the participants’ serial-recall performance on a scale ranging from − 6 (very distracting) to + 6 (very helpful) as a function of playback direction (forward, backward) and group (prospective judgments, control) in Experiment 1. The error bars represent the standard errors of the means.

A 2 × 2 mixed analysis of variance was calculated to test the effects of the within-subjects factor playback direction (forward, backward) and the between-subjects factor group (prospective judgments, control) on the global retrospective judgments (Fig. 4). Participants judged forward speech to have been significantly less distracting than backward speech, *F*(1, 254) = 19.54, *p* < 0.001, $\eta_p^2=0.07$. Participants in the prospective-judgments group judged speech to have been significantly less distracting than participants in the control group, *F*(1, 254) = 5.15, *p* = 0.024, $\eta_p^2=0.02$, but this effect was no longer statistically significant when participants were excluded who had failed to provide the correct answer in the catch trial or had reported technical issues or difficulties following the instructions. The main effects of playback direction and group were qualified by a significant interaction between playback direction and group, *F*(1, 254) = 9.09, *p* = 0.003, $\eta_p^2=0.03$. Participants in the prospective-judgments group incorrectly judged forward speech to have been significantly less distracting than backward speech, *F*(1, 133) = 21.87, *p* < 0.001, $\eta_p^2=0.14$, whereas participants in the control group correctly judged forward speech to have been as distracting as backward speech, *F*(1, 121) = 1.47, *p* = 0.228, $\eta_p^2=0.01$. Relative to the neutral midpoint of the scale, participants in the prospective-judgments group judged both forward speech, *t*(133) = − 6.76, *p* < 0.001, *d* = − 0.58, and backward speech to have been distracting, *t*(133) = − 13.88, *p* < 0.001, *d* = − 1.20. Participants in the control group also judged both forward speech, *t*(121) = − 13.21, *p* < 0.001, *d* = −1.20, and backward speech to have been distracting, *t*(121) = − 15.48, *p* < 0.001, *d* = − 1.40.

### Discussion

Replicating previous findings^29,37,39^, the results of Experiment 1 confirmed that forward speech was as distracting as backward speech. While the objective distraction effect of speech on serial-recall performance did not differ as a function of the playback direction of speech, forward speech gave rise to a higher subjective experience of processing fluency than backward speech. The main purpose of Experiment 1 was to test whether the stimulus-specific prospective judgments about the distracting effects of forward and backward speech would correspond to the objective distraction effects, favoring the direct-access account, or the experienced processing fluency, favoring the processing-fluency account. In line with the processing-fluency account, and inconsistent with the direct-access account, the stimulus-specific prospective judgments showed that forward speech was predicted not to be distracting relative to quiet, while backward speech was predicted to be distracting. This is parallel to the finding that people predicted backward music but not forward music to be distracting in a previous study upon which the present study was based^16^. Together, these results indicate that people rely on the processing-fluency heuristic when making stimulus-specific prospective judgments of distraction, leading to a metacognitive illusion irrespective of whether the distracting effects of instrumental music or the distracting effects of speech on serial-recall performance are to be predicted.

Participants who had not made stimulus-specific prospective judgments before the serial-recall task were able to correctly judge that forward speech had disrupted serial recall to the same degree as backward speech when providing global retrospective judgments of distraction at the end of the experiment. In contrast, participants who had made stimulus-specific prospective judgments before the serial-recall task failed to correctly judge that forward speech had disrupted serial recall to the same degree as backward speech. More precisely, even though forward speech was correctly judged to have been distracting relative to quiet, it was still judged to have been less distracting than backward speech. The requirement of providing stimulus-specific prospective judgments thus had a negative influence on the participants’ ability to retrospectively judge the distracting effects of forward and backward speech on their serial-recall performance.

## Experiment 2

Varying the playback direction of speech is a theoretically attractive manipulation of processing fluency because it keeps the number of auditory changes and the acoustic complexity of the speech—and thus the acoustic properties that are primarily responsible for auditory distraction in serial recall^1,2^—constant across both conditions (cf.^29^). However, varying the playback direction of speech is also a rather artificial manipulation of processing fluency, considering that speech played backward is a rare occurrence outside of the laboratory. Viewed from an ecological-validity perspective^24^, it may seem unsurprising that people fail to correctly predict the effects of a manipulation that is atypical of natural environments. Therefore, it is interesting to test whether a metacognitive illusion in stimulus-specific prospective judgments of distraction can be observed not only with forward and backward speech but also with a more ecologically valid manipulation of processing fluency.

In Experiment 2, we chose a more natural manipulation of processing fluency by presenting speech in the participants’ native language (German) or in a language foreign to them (Japanese), spoken by the same German-Japanese bilingual speaker. It has previously been demonstrated that native and foreign speech cause about the same amount of distraction in serial recall^37,41–43^. When differences between native and foreign speech were observed, these were small and in the direction of a somewhat more pronounced objective distraction effect in the native-speech condition than in the foreign-speech condition^44^. German speech^56 ^and Japanese speech^57^ are linguistically unrelated languages and their phonetic and prosodic properties differ substantially. Native speech should lead to a higher subjective experience of processing fluency than foreign speech. If participants rely on the experienced processing fluency when making stimulus-specific prospective judgments of distraction, native speech should be predicted to be less distracting than foreign speech. The objective distraction effect of speech on serial-recall performance, by contrast, should not differ as a function of whether native or foreign speech is presented.

### Method

#### Participants

Participants who had not taken part in Experiment 1 were recruited in the same way as in Experiment 1. Of the 332 participants who had passed the audio check, 63 participants did not complete the experiment or withdrew their consent to the use of their data, one participant was under 18 years old and 11 participants reported studying or having studied Psychology. Data integrity checks led to the exclusion of one further data set. Furthermore, one data set was excluded because the participant indicated that Japanese was their native language. As a consequence, 77 data sets could not be included into the final analysis. Based on the response in the catch trial or responses to the post-experiment questions, it would have been possible to exclude data sets of a further 45 participants. Following a recommendation of Elliott et al.^46^, we included these data sets into the final analysis. There was one case in which this decision affected the statistical conclusions which is reported below. The final sample, characterized by diversified levels of education, consisted of *N* = 255 participants (112 female, 141 male, 2 nonbinary) with a mean age of 35 (*SD* = 14) years. All participants were proficient German speakers; 241 indicated that German was their native language (14 indicated that a language other than German or Japanese was their native language) and 235 reported not to speak Japanese (20 reported to speak a little Japanese). Participants were randomly assigned to either the prospective-judgments group (*n* = 133) or the control group (*n *= 122). A sensitivity analysis^47^ showed that, given a sample size of *N* = 255 and α = 0.05, a main effect of the language of speech on the objective distraction effect of the size $\eta_p^2=0.05$ could be detected with a statistical power of 1 − β = 0.95.

#### Materials

The sounds consisted of 12 speech sequences that were highly similar to those used in Experiment 1. The Japanese speech sequences were direct translations of the German speech sequences. The German and Japanese speech sequences were spoken by the same male German-Japanese bilingual speaker. Each sound lasted eight seconds. Using Amadeus Pro (HairerSoft, Version 2.8.9), the sounds were normalized to peak amplitude. Whereas length and content of the German and Japanese speech sequences were the same, German^56 ^and Japanese^57^ are linguistically unrelated languages and their speech differs in global and local phonetic and phonological properties such as melody, rhythm, intonation, syllable structure and speech sounds. Japanese speech should evoke a lower subjective experience of processing fluency than German speech because it runs counter to conventional auditory processing patterns to which German-speaking participants are accustomed.

A separate validation study with different participants was performed to test whether the manipulation of processing fluency was successful. After having applied the same exclusion criteria as described above, data sets of *N* = 107 participants (42 female, 65 male) with a mean age of 41 (*SD *= 16) years were included into the final analysis. All participants were proficient German speakers; 103 indicated that German was their native language (4 indicated that a language other than German or Japanese was their native language) and 97 reported not to speak Japanese (10 reported to speak a little Japanese). The 24 sounds—12 speech sequences with native speech, 12 speech sequences with foreign speech, presented in a random order—were rated one by one. The same single-item processing-fluency scale^51^ as in the validation study of Experiment 1 was used to measure the subjective experience of processing fluency. Using the speech sequences as the units of analysis, native speech (*M* = 4.72, *SD* = 0.27) was rated to be significantly easier to listen to than foreign speech (*M* = 1.92, *SD* = 0.13), *F*(1, 22) = 1035.70, *p* < 0.001, $\eta_p^2=0.98$, which leads to the conclusion that processing fluency was manipulated successfully.

#### Design and procedure

Design and procedure were the same as in Experiment 1 with the following exceptions: Each participant in the prospective-judgments group assessed six native-speech sequences and six foreign-speech sequences in the prospective-judgments task. These speech sequences were randomly selected from a pool of 12 native-speech sequences and 12 foreign-speech sequences. The 18 experimental trials of the serial-recall task included six distractor trials with native speech, six distractor trials with foreign speech and six quiet control trials in an order that was randomized at an individual level. For each participant in the prospective-judgments group, the six native-speech sequences and the six foreign-speech sequences that had not been presented in the prospective-judgments task were used as distractor sequences in the serial-recall task. For each participant in the control group, six native-speech sequences and six foreign-speech sequences were randomly selected from the pool of 12 native-speech sequences and 12 foreign-speech sequences, respectively. The median duration of the experiment was 17 minutes in the prospective-judgments group and 13 minutes in the control group.

### Results

#### Stimulus-specific prospective judgments of distraction

**Figure 5:**
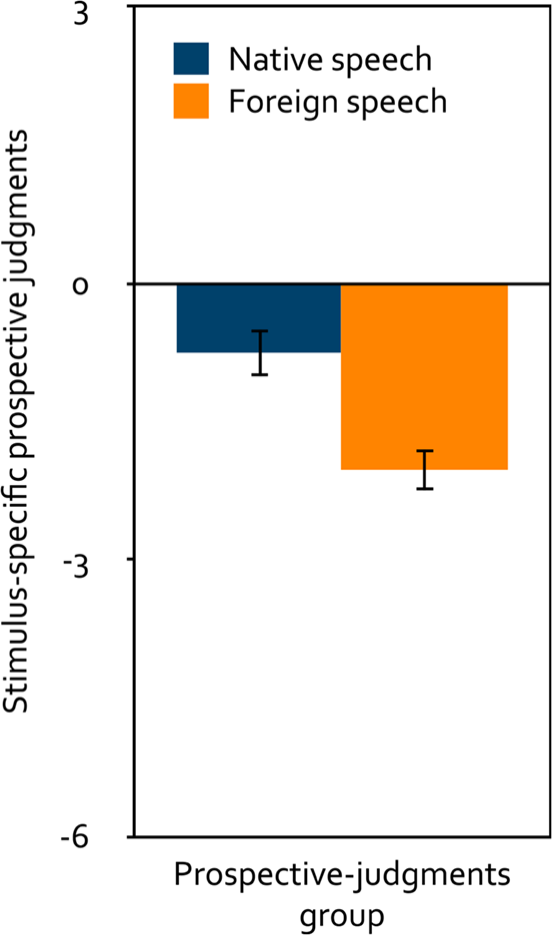
Mean stimulus-specific prospective judgments about the distracting effect of speech on serial-recall performance on a scale ranging from − 6 (very distracting) to + 6 (very helpful) as a function of language (native, foreign) in Experiment 2. The error bars represent the standard errors of the means.

A repeated-measures analysis of variance was calculated to test the effect of the within-subjects factor language (native, foreign) on the stimulus-specific prospective judgments (Fig. 5). Participants in the prospective-judgments group predicted native speech to be significantly less distracting than foreign speech, *F*(1, 132) = 28.60, *p* < 0.001, $\eta_p^2=0.18$. Relative to the neutral midpoint of the scale, both native speech, *t*(132) = − 3.03, *p* = 0.003, *d* = − 0.26, and foreign speech were predicted to be distracting, *t*(132) = − 9.20, *p* < 0.001, *d* = − 0.80.

#### Objective distraction effects

The objective distraction effects of speech on serial-recall performance were calculated for each participant by subtracting, from the mean number of digits per trial that were recalled at the correct serial position in each of the two variants of the distractor trials (with native or foreign speech), the mean number of digits per trial that were recalled at the correct serial position in the quiet control trials. The serial-recall results that formed the basis for calculating the objective distraction effects, averaged across participants in each group, are reported in Table 2.

**Table 2:** Mean number of digits per trial that were recalled correctly in each of the two variants of the distractor trials (native speech, foreign speech) and in the quiet control trials of the serial-recall task as a function of group (prospective judgments, control) in Experiment 2. Eight different digits were presented per trial. A strict serial-recall criterion was used in that only digits reproduced at the correct serial position were scored as correct. The values in parentheses represent the standard errors of the means.

	Prospective-judgments group		Control group	
Native speech	4.38	(0.19)	4.46	(0.19)
Foreign speech	4.46	(0.19)	4.56	(0.19)
Quiet control	4.88	(0.18)	5.31	(0.18)

**Figure 6:**
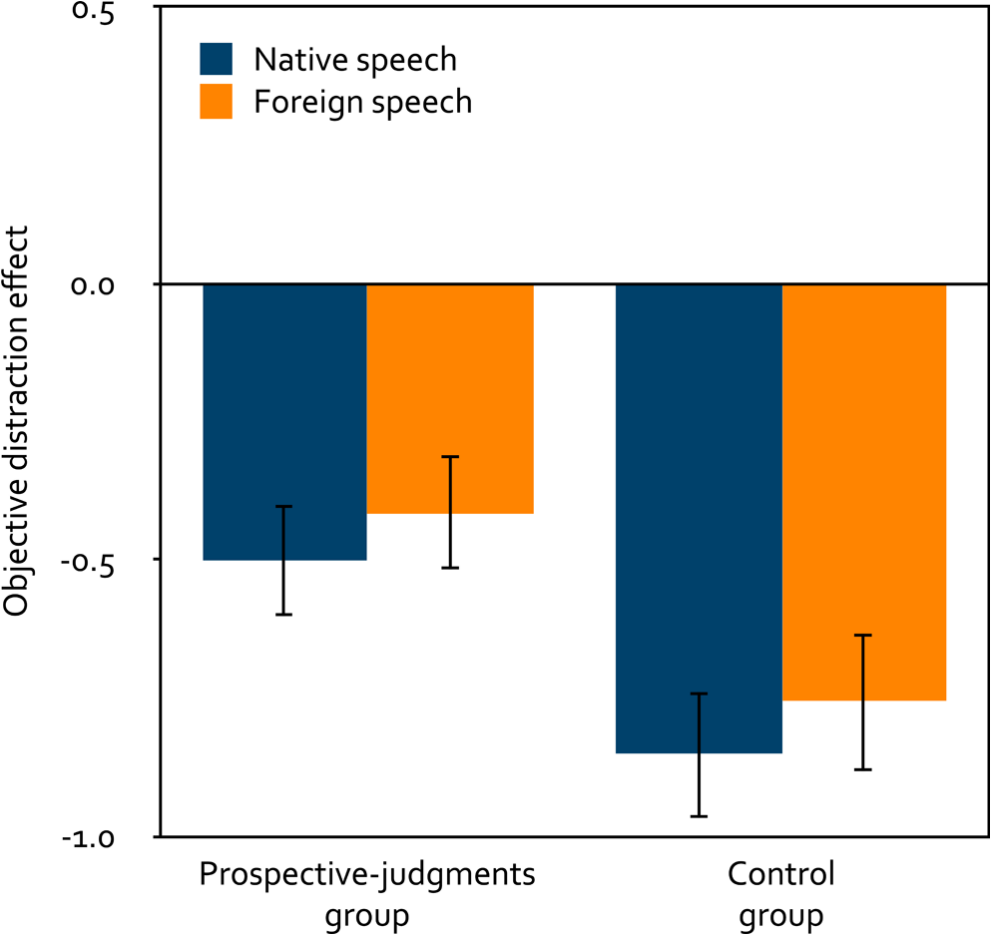
Mean objective distraction effect of speech on serial-recall performance as a function of language (native, foreign) and group (prospective judgments, control) in Experiment 2. The objective distraction effects were calculated by subtracting, from the mean number of digits per trial recalled correctly in the distractor trials, the mean number of digits per trial recalled correctly in the quiet control trials. Negative values thus reflect a distracting effect of the speech (positive values would reflect a helpful effect). The error bars represent the standard errors of the means.

A 2 × 2 mixed analysis of variance was calculated to test the effects of the within-subjects factor language (native, foreign) and the between-subjects factor group (prospective judgments, control) on the objective distraction effect of speech on serial-recall performance (Fig. 6). Native speech disrupted serial recall just as much as foreign speech, *F*(1, 253) = 1.82, *p* = 0.179, $\eta_p^2=0.01$. Participants in the prospective-judgments group were less distracted by the speech than participants in the control group, *F*(1, 253) = 6.09, *p* = 0.014, $\eta_p^2=0.02$, but this effect was no longer statistically significant when participants were excluded who had failed to provide the correct answer in the catch trial or had reported technical issues or difficulties following the instructions. Group did not interact with language, *F*(1, 253) < 0.01, *p* = 0.954, $\eta_p^2< 0.01$. In the prospective-judgments group, both native speech, *t*(132) = − 4.94, *p* < 0.001, *d* = − 0.43, and foreign speech, *t*(132) = − 4.02, *p* < 0.001, *d* = − 0.35, had a distracting effect on serial-recall performance relative to quiet. Likewise, in the control group, both native speech, *t*(121) = − 7.49, *p* < 0.001, *d* = − 0.68, and foreign speech, *t*(121) = − 6.16, *p* < 0.001, *d* = − 0.56, had a distracting effect on serial-recall performance relative to quiet.

#### Global retrospective judgments of distraction

**Figure 7:**
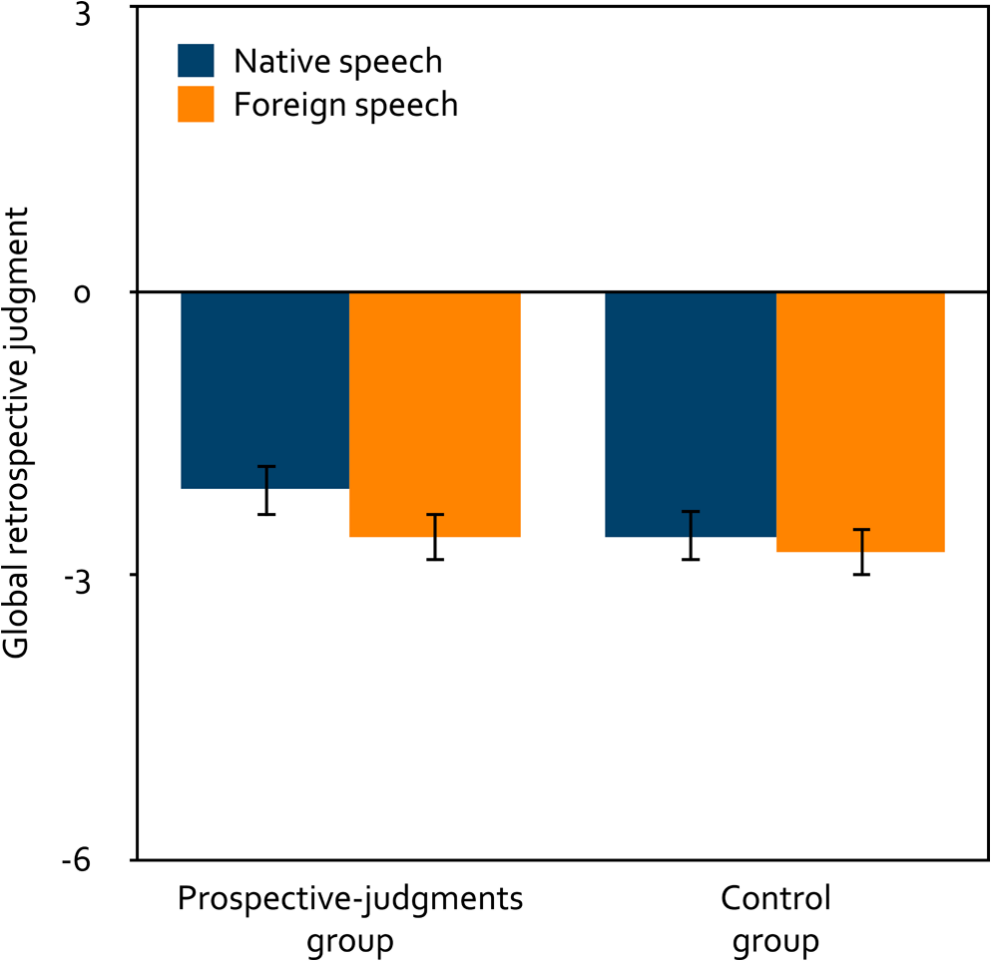
Mean global retrospective judgment about the distracting effect of speech on the participants’ serial-recall performance on a scale ranging from − 6 (very distracting) to + 6 (very helpful) as a function of language (native, foreign) and group (prospective judgments, control) in Experiment 2. The error bars represent the standard errors of the means.

A 2 × 2 mixed analysis of variance was calculated to test the effects of the within-subjects factor language (native, foreign) and the between-subjects factor group (prospective judgments, control) on the global retrospective judgments (Fig. 7). Participants correctly judged that native speech had disrupted serial recall just as much as foreign speech, *F*(1, 253) = 3.70, *p* = 0.056, $\eta_p^2=0.01$. Whether or not stimulus-specific prospective judgments had been collected before the serial-recall task did not affect the global retrospective judgments, *F*(1, 253) = 0.94, *p* = 0.332, $\eta_p^2< 0.01$, and did not interact with language, *F*(1, 253) = 0.81, *p* = 0.370, $\eta_p^2< 0.01$. Relative to the neutral midpoint of the scale, participants in the prospective-judgments group judged both native speech, *t*(132) = − 8.07, *p* < 0.001, *d* = − 0.70, and foreign speech to have been distracting, *t*(132) = − 9.87, *p* < 0.001, *d* = − 0.86. Participants in the control group also judged both native speech, *t*(121) = − 9.21, *p* < 0.001, *d* = − 0.83, and foreign speech to have been distracting, *t*(121) = − 11.26, *p* < 0.001, *d* = − 1.02.

### Discussion

Replicating previous findings^37,41–43^, speech disrupted serial recall independently of whether it was presented in the participants’ native language or in a language foreign to them, presumably because the number of auditory changes and the acoustic complexity of the speech—that are primarily responsible for auditory distraction in serial recall^1,2^—were comparable for native and foreign speech. However, native speech gave rise to a higher subjective experience of processing fluency than foreign speech. The stimulus-specific prospective judgments showed that native speech was predicted to be less distracting than foreign speech. The stimulus-specific prospective judgments thus corresponded to the experienced processing fluency and not to the objective distraction effects, favoring the processing-fluency account over the direct-access account. This replicates conceptually the findings of Experiment 1 and demonstrates that the metacognitive illusion in stimulus-specific prospective judgments of distraction by speech persists when an ecologically valid manipulation of processing fluency is used.

In those participants who had provided stimulus-specific prospective judgments of distraction, the objective distraction effects of speech on serial-recall performance were generally reduced. However, this effect was only associated with a small sample effect size and was no longer statistically significant when participants were excluded who had failed to provide the correct answer in the catch trial or had reported technical issues or difficulties following the instructions. Together with the fact that providing stimulus-specific prospective judgments of distraction had no effect on the objective distraction effect in Experiment 1 and in a previous study using instrumental music as the stimulus material^16^, it is reasonable to conclude that the requirement of providing stimulus-specific prospective judgments of distraction before the serial-recall task does not robustly modulate the objective distraction effects of sounds on serial-recall performance.

When providing global retrospective judgments of distraction, participants were able to correctly judge that native speech had disrupted serial recall to the same degree as foreign speech. Having experienced the distracting effects of the different types of speech on their serial-recall performance firsthand may have provided participants with cues that prevented them from falling victim to a metacognitive illusion in their global retrospective judgments of distraction.

## General discussion

The main purpose of the present study was to test how people arrive at stimulus-specific prospective judgments with which they predict the distracting effects of speech on cognitive performance. The direct-access account implies that people have direct metacognitive access to the cognitive effects of sounds that determine distraction. The processing-fluency account implies that people rely on the processing-fluency heuristic to predict the distracting effects of sounds on cognitive performance. To test the direct-access account against the processing-fluency account, we focused on situations in which these two accounts lead to different predictions.

In Experiment 1, we manipulated processing fluency by varying the playback direction of speech sequences. Replicating previous findings^29,37,39^, forward and backward speech disrupted serial recall to the same degree, presumably because the acoustic properties that are primarily responsible for auditory distraction in serial recall, such as the number of auditory changes and the acoustic complexity of the speech^1,2^, remain unaffected by the playback direction of speech. Specifically, the number of auditory changes should be equivalent in forward and backward speech, implying that the processing of these changes should cause the same amount of distraction in serial recall according to dominant accounts of auditory distraction such as the interference-by-process account^30–33 ^and the attentional account^29,34–36^. Although, as expected, the playback direction of speech did not affect the objective distraction effect of speech on serial-recall performance, the playback direction of speech had a strong effect on the ratings of the subjective experience of processing fluency, confirming that forward speech gives rise to a higher subjective experience of processing fluency than backward speech. According to the direct-access account, the stimulus-specific prospective judgments should reflect that the playback direction of speech does not affect the objective distraction effect of speech on serial-recall performance. According to the processing-fluency account, the stimulus-specific prospective judgments should reflect the experienced processing fluency of the speech. In line with the processing-fluency account, and inconsistent with the direct-access account, participants predicted only backward speech, but not forward speech, to be distracting. This provides evidence of a metacognitive illusion in stimulus-specific prospective judgments of distraction by speech.

The finding that participants failed to correctly predict the distracting effects of forward and backward speech on serial-recall performance is completely parallel to the finding that participants failed to correctly predict the distracting effects of forward and backward music on serial-recall performance observed in a previous study^16^. In that study, forward and backward music caused the same amount of distraction in serial recall, consistent with previous research^28,29^. However, forward music was associated with a higher subjective experience of processing fluency than backward music. In line with the assumption that people heuristically rely on processing fluency when predicting the distracting effects of music on serial-recall performance, participants predicted only backward music, but not forward music, to be distracting. The results of the experiments reported here extend these findings by demonstrating that the failure to correctly predict the distracting effects of sounds on cognitive performance is not unique to instrumental music. Instead, the findings reported here indicate that the processing-fluency heuristic leads to a metacognitive illusion in stimulus-specific prospective judgments of distraction whenever objective distraction effects and subjective experiences of processing fluency are in opposition to each other, irrespective of whether the distracting effects of instrumental music or the distracting effects of speech on cognitive performance are to be predicted. The conclusion that stimulus-specific prospective judgments of distraction are based on the processing-fluency heuristic aligns with our understanding of how people arrive at other metacognitive judgments such as judgments of learning^58^. Evidence suggests that people frequently rely on the processing-fluency heuristic when making stimulus-specific prospective judgments of learning (e.g.,^3,5,6,9,20,21^). For instance, stimuli that give rise to a strong subjective experience of processing fluency are predicted to be easier to learn and to remember than stimuli that evoke a weak subjective experience of processing fluency^59^. The findings reported here suggest that stimulus-specific prospective judgments of distraction must be included in the list of metacognitive judgments that are based on the processing-fluency heuristic and are thus subject to metacognitive illusions.

A limitation of Experiment 1 that is shared by a previous study from our laboratory^16^ is that the experimental test of the processing-fluency account relied on a rather artificial manipulation of processing fluency in that backward speech or backward music are rarely encountered in everyday life. This raises the question of whether a metacognitive illusion in stimulus-specific prospective judgments of distraction can also be obtained with a more ecologically valid manipulation of processing fluency.

In Experiment 2, we therefore examined how people predict the distracting effects of native and foreign speech on serial-recall performance. Consistent with previous findings^37,41–43^, native and foreign speech caused the same amount of distraction in serial recall. However, native speech was experienced more fluently than foreign speech. Following the ratings of the subjective experience of processing fluency, participants predicted native speech to be less distracting than foreign speech. The results of Experiment 2 thus show a metacognitive illusion with a more natural manipulation of processing fluency than that used in Experiment 1.

To summarize, metacognitive illusions in stimulus-specific prospective judgments of distraction, due to the use of the processing-fluency heuristic, have been obtained with forward and backward music^16^, forward and backward speech (Experiment 1) and native and foreign speech (Experiment 2). Given this, it is easy to generate additional hypotheses about specific situations in which the processing-fluency heuristic can be expected to lead to metacognitive illusions. For example, speech in one’s native language should be experienced to be easier to listen to than speech in a second language. Therefore, the processing-fluency account allows to derive the hypothesis that people will incorrectly predict native-language speech to be less distracting than second-language speech, with the difference between the two types of speech in stimulus-specific prospective judgments of distraction depending on how fluent people are in the second language. This shows that the processing-fluency account is productive in that it makes it easy to derive novel hypotheses that can guide future research. However, even though the available experiments demonstrate powerful metacognitive illusions in stimulus-specific prospective judgments of distraction by instrumental music or speech, it is important to note that these experiments were deliberately designed to create situations in which processing fluency is an invalid cue for predicting the distracting effects of speech on cognitive performance. Identifying situations in which the processing-fluency heuristic leads to incorrect stimulus-specific prospective judgments of distraction does not imply that the processing-fluency heuristic lacks adaptive value in everyday life (cf.^24^). In fact, it has already been demonstrated that relying on the processing-fluency heuristic can also lead to correct stimulus-specific prospective judgments of distraction. For instance, people are aware of the fact that repeated speech is less distracting than deviating speech which is, in turn, less distracting than changing speech^14,15^. Given that repeated exposure to an identical stimulus is known to enhance the subjective experience of fluency associated with the processing of that stimulus^60^, these results indicate that conditions exist in which the processing-fluency heuristic leads to valid stimulus-specific prospective judgments of distraction. Even so, the present study shows that there is no direct correspondence between processing fluency and objective distraction.

After participants in Experiment 2 had experienced the distracting effects of native and foreign speech on their serial-recall performance firsthand, they correctly judged native speech to have been as distracting as foreign speech when providing global retrospective judgments of distraction. A similar pattern was observed in Experiment 1 in which the global retrospective judgments correctly reflected the fact that forward speech had disrupted serial recall to the same degree as backward speech, even though this finding was only obtained when participants had not provided stimulus-specific prospective judgments of distraction before the serial-recall task. These findings suggest that, with firsthand experience of performing the serial-recall task while ignoring the different types of speech, participants gain access to comparatively valid cues to judge the distracting effects of the different types of speech on their serial-recall performance. These cues may have helped participants to correctly represent the degree to which they were distracted by the different types of speech. This fits with previous findings showing that retrospective judgments reflect the relative distracting effects of different types of sound better than prospective judgments^25^. Therefore, firsthand experience with the distracting effects of the different types of speech on the participants’ serial-recall performance may prevent a metacognitive illusion, leading to comparatively accurate global retrospective judgments of distraction.

A limitation of the present study is that it was not designed to, and thus did not yield results that can be used to, differentiate between dominant accounts of auditory distraction such as the interference-by-process account^30–33 ^and the attentional account^29,34–36^. Instead, the present study served to test accounts of how people arrive at stimulus-specific prospective judgments about the distracting effects of sounds on cognitive performance. The results obtained in the present study confirm the account according to which people rely on the processing-fluency heuristic to predict the distracting effects of sounds on cognitive performance. The results disconfirm the account according to which people have direct access to the cognitive effects of sounds that determine distraction. Given this focus, the present study does not address issues such as whether the distracting effects of sounds are driven by automatic or controlled processes^15,48,61^, are domain-specific or domain-general^36,62–64 ^or depend on general processing resources^49,65^. Past controversies have primarily focused on these issues rather than on what drives the metacognition of auditory distraction, which is a relatively recent aspect of research in auditory distraction^14,17^. By providing novel insights into the metacognition of auditory distraction, the present study thus makes its contribution by expanding rather than by confirming or disconfirming traditional accounts of auditory distraction.

Furthermore, the present experiments were designed to test whether a metacognitive illusion exists in the stimulus-specific prospective judgments about the distracting effects of speech on cognitive performance. Now that we have established *that* such a metacognitive illusion exists, future research should address the question of *why *this metacognitive illusion exists. A promising explanation, adapted from Reber et al.^22^, is that subjective fluency may be experienced at different levels of processing, which need not all be equally relevant for objective distraction in serial recall. For example, as in the processing of pseudowords^66,67^, participants may experience difficulties processing backward speech and foreign speech at a conceptual level and use the resulting subjective experience of relatively disfluent processing as a cue that the speech will be harder to ignore. However, evidence suggests that the objective distraction effect of speech on serial-recall performance is largely independent of the conceptual characteristics of the to-be-ignored sounds^16,29,37–43^. Future research should test this hypothesis systematically to determine whether it proves to be a successful explanation for the discrepancy between stimulus-specific prospective judgments of distraction and objective distraction effects of sounds on cognitive performance.

In sum, the present study served to examine how people arrive at stimulus-specific prospective judgments about the distracting effects of speech on cognitive performance. The results indicate that people are prone to a metacognitive illusion when predicting the distracting effects of speech. Even though forward speech and native speech disrupted serial recall to the same degree as backward speech and foreign speech, respectively, participants incorrectly predicted the more fluently experienced forward speech and native speech to be less distracting than backward speech and foreign speech. These results are compatible with the assumption that people predict the distracting effects of individual speech sequences on cognitive performance by relying on the processing-fluency heuristic. When predicting the distracting effects of sounds on cognitive performance, people should thus be aware of the fact that the heuristics used to arrive at stimulus-specific prospective judgments of distraction may often be correct but may also cause systematic biases. Firsthand experience with the distracting effects of the different types of speech on the participants’ cognitive performance, by contrast, leads to comparatively accurate global retrospective judgments of distraction.

## Data Availability

The data of Experiments 1 and 2 as well as the German speech sequences used in Experiment 1 and the German and Japanese speech sequences used in Experiment 2 (together with English translations) are available at the project page of the Open Science Framework, https://osf.io/wqgjf/.
